# Pumpless Extracorporeal Hemadsorption Technique (pEHAT): A Proof-of-Concept Animal Study

**DOI:** 10.3390/jcm11226815

**Published:** 2022-11-18

**Authors:** Mascha O. Fiedler, Ralf M. Muellenbach, Caroline Rolfes, Christopher Lotz, Felix Nickel, Beat P. Müller-Stich, Alexander Supady, Philipp M. Lepper, Markus A. Weigand, Patrick Meybohm, Armin Kalenka, Christian Reyher

**Affiliations:** 1 Department of Anesthesiology, Heidelberg University Hospital, 69120 Heidelberg, Germany; 2 Department of Anesthesiology, Critical Care Medicine, Emergency Medicine and Pain Therapy, Campus Kassel of the University of Southampton, 34125 Kassel, Germany; 3 Department of Anaesthesiology, Intensive Care, Emergency and Pain Medicine, University Hospital Wuerzburg, 97080 Wuerzburg, Germany; 4 Department of General, Visceral and Transplantation Surgery, Heidelberg University Hospital, 69120 Heidelberg, Germany; 5 Faculty of Medicine, University of Freiburg, 79106 Freiburg, Germany; 6 Department of Cardiology and Angiology I, Heart Center, University of Freiburg, 79106 Freiburg, Germany; 7 Heidelberg Institute of Global Health, University of Heidelberg, 69117 Heidelberg, Germany; 8 Department of Internal Medicine V—Pneumology, Allergology and Critical Care Medicine, University Medical Centre, Saarland University, 66424 Homburg, Germany; 9 Department of Anesthesiology and Intensive Care Medicine, Hospital Bergstrasse, 64646 Heppenheim, Germany

**Keywords:** blood purification, extracorporeal hemadsorption, cytokines, adsorption, animal model, immunosorbents, septic shock, endotoxin, extracorporeal techniques in hemadsorption therapy, arteriovenous extracorporeal hemadsorption technique

## Abstract

**Background**: Extracorporeal hemadsorption eliminates proinflammatory mediators in critically ill patients with hyperinflammation. The use of a pumpless extracorporeal hemadsorption technique allows its early usage prior to organ failure and the need for an additional medical device. In our animal model, we investigated the feasibility of pumpless extracorporeal hemadsorption over a wide range of mean arterial pressures (MAP). **Methods**: An arteriovenous shunt between the femoral artery and femoral vein was established in eight pigs. The hemadsorption devices were inserted into the shunt circulation; four pigs received CytoSorb^®^ and four Oxiris^®^ hemadsorbers. Extracorporeal blood flow was measured in a range between mean arterial pressures of 45–85 mmHg. Mean arterial pressures were preset using intravenous infusions of noradrenaline, urapidil, or increased sedatives. **Results**: Extracorporeal blood flows remained well above the minimum flows recommended by the manufacturers throughout all MAP steps for both devices. Linear regression resulted in CytoSorb^®^ blood flow [mL/min] = 4.226 × MAP [mmHg] − 3.496 (R-square 0.8133) and Oxiris^®^ blood flow [mL/min] = 3.267 × MAP [mmHg] + 57.63 (R-square 0.8708), respectively. **Conclusion**: Arteriovenous pumpless extracorporeal hemadsorption resulted in sufficient blood flows through both the CytoSorb^®^ and Oxiris^®^ devices over a wide range of mean arterial blood pressures and is likely an intriguing therapeutic option in the early phase of septic shock or hyperinflammatory syndromes.

## 1. Background

Extracorporeal hemadsorption (HA) is a blood purification technique utilized in critically ill patients [1]. Sorbent-containing cartridges eliminate cytokines, myoglobin, bilirubin, toxins, and various drugs from the bloodstream [2,3]. It is often used in states of septic shock with hyperinflammation to reduce proinflammatory mediators. Current evidence supporting the use of extracorporeal HA techniques is not conclusive; however, the available data suggest that its use is safe and is most effective in the early phase of septic shock, i.e., within the first 24 h of the dysregulated host response [4,5]. Hemadsorption devices are usually integrated with an extracorporeal circuit such as continuous renal replacement therapy (CRRT) or extracorporeal membrane oxygenation (ECMO). Thus, the clinical application of HA often is postponed until the presence of organ failure, e.g., acute renal or lung failure, with the need for CRRT or ECMO therapy [6,7,8]. This is usually performed in close collaboration with nephrologists. Recently, a new pumpless extracorporeal hemadsorption technique (pEHAT) was described as an HA technique independent of an additional medical device [9]. This pumpless arteriovenous use allows hemadsorber therapy independent of a CRRT or ECMO therapy and could help to prevent severe organ dysfunction. However, effective blood purification requires a constant blood flow > 100 mL/min through the hemadsorber [10]. In pEHAT, blood flow is driven by systemic mean atrial pressure (MAP). The aim of this animal study was to investigate the feasibility and hemodynamic characteristics of two different hemadsorption systems over a wide range of MAPs. Our study should be the basis for future research using pEHAT in a clinical setting.

## 2. Methods

### 2.1. Animal Preparation

The study was approved by the responsible committee for animal research (Regierungspräsidium Karlsruhe 35-9185.81/G-109/20). Eight pigs (48.3 ± 5.1 kg body weight) were included.

After overnight fasting with free access to water, female domestic pigs were anaesthetized intramuscularly in combination with 7 mg/kg Azaperon (Stresnil^®^, Lilly, Bad Homburg, Germany), 2.5 mg/kg S-Ketamin (Pfizer Pharma, Berlin, Germany), and 0.3 mg/kg Midazolam (Midazolam, Hameln Pharma, Hameln, Germany).

Pigs were intubated and mechanically ventilated with a Primus^®^ ventilator (Drägerwerke, Lübeck, Germany) using an inspiratory oxygen concentration (F_i_O_2_) of 0.4 in a volume-controlled modus. A tidal volume of 8 mL/kg bodyweight (bw), an inspiration/expiration ratio of 1:2, and a PEEP of 5 cmH_2_O were provided. Anesthesia was maintained by continuous infusion of 6 mg/kg/h S-Ketamin and 3.6 mg/kg/h Midazolam. There was no use of neuromuscular blockers. Depth of anesthesia was regularly assessed by absence of spontaneous breathing efforts and lack of muscle tone.

### 2.2. Instrumentation

An arterial catheter was inserted with ultrasound guidance (VScan^®^, GE Ultrasound, Horten, Norway) into the left internal carotid artery to monitor systemic arterial pressure and to draw arterial blood gas samples. A pulmonary arterial catheter was inserted via the right external jugular vein to measure pulmonary arterial pressure (PAP), central venous pressure (CVP), and continuous cardiac output (CCO) (Vigilance II^®^, Edwards Lifesciences, Irvine, CA, USA).

An arteriovenous shunt was established in eight pigs between the femoral artery and femoral vein. In order to limit the risks of arterial ischemia while concomitantly maintaining sufficient blood flow through the HA device, a small 10 Fr arterial cannula (Bio-Medicus^™^, Medtronic, Minneapolis, MN, USA) was placed as the arterial line. The distal extremity of the animals was monitored only macroscopically. A 14 French venous cannula (Bio-Medicus^™^, Medtronic, Minneapolis, MN, USA) was utilized for venous return to allow the most unresisted outflow possible. All catheters were emplaced after skin disinfection under sterile conditions. In all pigs, an extracorporeal HA device was inserted into the shunt circulation with blood flowing from the femoral artery through the adsorber into the femoral vein. The priming volume of the extracorporeal circuit and cartridge was approximately 180–200 mL crystalloid solution.

The animals were randomly assigned to one of the following treatment groups (n = 4/group):

1. CytoSorb^®^ HA (CytoSorbents Inc., Monmouth Junction, NJ, USA).
2. Oxiris^®^ HA (Baxter, Meyzieu, France).

CytoSorb^®^ is a hemadsorption device and Oxiris^®^ has a double-function mechanism: hemadsorption and -perfusion. Both of them are useable in combination with hemodialysis (HD) or continuous venovenous hemofiltration (CVVH). The CytoSorb^®^ consists of porous polymer beads and it has the largest surface area compared to other comparable devices. Oxiris^®^ has an AN69-based membrane [11], and the surface is treated with polyethyleneimine and grafted with heparin. One advantage of using Oxiris^®^ is a lower risk of thrombogenicity by adsorbing antithrombin-III from the blood. The indication for both methods is the removal of inflammatory cytokines, such as in severe sepsis and septic shock.

**Figure 1:**
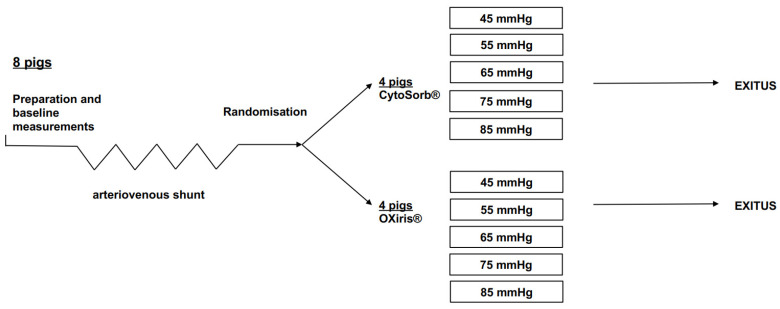
Experimental timeline. After baseline preparation, an arteriovenous shunt was established in eight pigs between the femoral artery and femoral vein. In all pigs, an extracorporeal HA device was inserted into the shunt circulation with blood flowing from the femoral artery through the adsorber into the femoral vein. The animals were randomly assigned to one of the two treatment groups (n = 4/group). MAP was varied between 45 mmHg and 85 mmHg in incremental steps of 10 mmHg every 20 min using continuous intravenous infusions of noradrenaline or urapidil.

Since mean arterial pressure (MAP) is one of the major determinants of blood flow through the HA device, MAP was varied between 45 mmHg and 85 mmHg in incremental steps of 10 mmHg every 20 min using continuous intravenous infusions of noradrenaline or urapidil (Figure 1). Pre- and post-HA pressures, cardiac output (CO), and shunt blood flows (Medicovation RealFlow, Gladbeck, Germany) were continuously recorded. During the experimental setting, full anticoagulation was achieved by an intravenous bolus of 50 units/kg heparin, followed by 20 units/kg/h. At the end of the experimental protocol, the pigs were euthanized with potassium chloride.

### 2.3. Data Analysis

Data were analyzed with repeated measures analysis of variance followed by post hoc Bonferroni’s multiple comparison test using Prism 5 for Mac OS X (GraphPad Software, San Diego, CA, USA). Changes were considered statistically significant when the two-sided *p*-value was less than 0.05. All data are expressed as mean ± standard deviation (SD).

## 3. Results

A total of eight pigs were instrumented to obtain four successful experiments in each experimental group. In all animals the experimental protocol was completed without minor or major complications.

### 3.1. Systemic Hemodynamics

Hemodynamic parameters over the course of the experimental protocol are shown in Table 1. Significant changes (*p* < 0.05) in heart rate were observed in pigs treated with CytoSorb^®^ hemadsorption at MAP 85 mmHg (vs. MAP 45, MAP 55, and MAP 65 mmHg, respectively) in the form of a mild tachycardia (103 ± 6.4 bpm). This was not observed in pigs treated with the Oxiris^®^ device. On the other hand, pigs treated with Oxiris^®^ had significant changes (*p* < 0.05) in PAP (baseline vs. MAP 45 and MAP 55; MAP 45 vs. MAP 65; MAP 55 and MAP 65 vs. MAP 85 mmHg, respectively) and CVP (MAP 45 vs. MAP 85 mmHg, respectively). The highest PAP value was 26 ± 2.2 mmHg at MAP 85 mmHg. Significant changes in PAP were not present during CytoSorb^®^ treatment. Regardless of the utilized device, cardiac index (CI) remained within normal values without significant changes throughout the experimental protocol.

### 3.2. Hemadsorber Blood Flow

**Figure 2:**
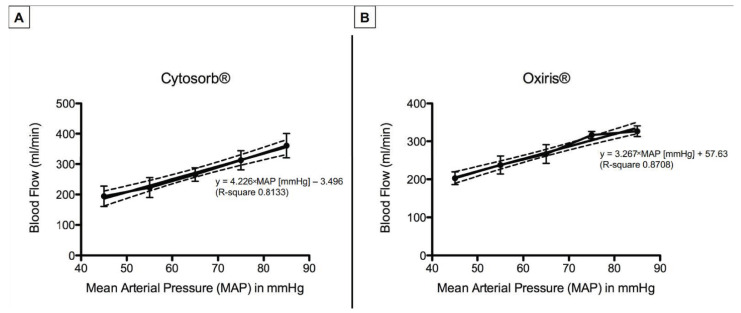
Blood flow through the CytoSorb^®^ (**A**) or Oxiris^®^ hemadsorber (**B**). Mean blood flow through both devices linearly increased with stepwise increases in mean arterial pressure. Each figure depicts mean blood flow values ± SD, as well as the linear regression line and their 95% confidence band of the best-fit line (scattered lines).

Absolute values of mean blood flows throughout the experiment are shown in Table 2. Maximum blood flows were achieved at MAP of 85 mmHg with 360.7 ± 39.9 mL/min for CytoSorb^®^ and 326.6 ± 14.1 mL/min for Oxiris^®^. Blood flows remained well above the optimum flow recommended by the manufacturers of >100 mL/min. When directly comparing the CytoSorb^®^ and Oxiris^®^ devices, there were no significant differences in mean blood flow through the devices during all MAP steps of the experimental protocol. Blood flows through the CytoSorb^®^ and Oxiris^®^ hemadsorber linearly increased with increasing mean blood pressures. Considering the impact of MAP on changes in blood flow, linear regression showed blood flow [mL/min] = 4.226 × MAP [mmHg] − 3.496 (R-square 0.8133) for CytoSorb^®^ and blood flow [mL/min] = 3.267 × MAP [mmHg] + 57.63 (R-square 0.8708) for the Oxiris^®^ hemadsorber, respectively (Figure 2). This emphasizes the linear interdependence of MAP and hemadsorber blood flow for both devices.

### 3.3. Pre- and Post-Hemadsorber Pressure

**Figure 3:**
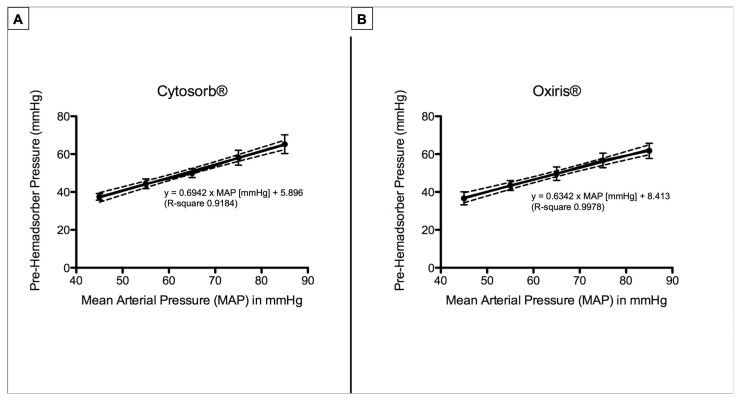
Pre-hemadsorber pressures for the CytoSorb^®^ (**A**) and Oxiris^®^ (**B**) devices. Mean pre-hemadsorber pressures linearly increased with stepwise increases in mean arterial pressure. Each figure depicts mean pre-hemadsorber pressure ± SD, as well as the linear regression line and their 95% confidence band of the best-fit line (scattered lines).

Absolute values of mean pre- and post-hemadsorber pressures throughout the experiment are shown in Table 2. Pre-absorber pressures significantly differed between all experimental steps (*p* < 0.0001). Linear regression of mean arterial pressure and pre-hemadsorber pressure resulted in pre-hemadsorber pressure [mmHg] = 0.6942 × MAP [mmHg] + 5.896 (R-square 0.9184) for CytoSorb^®^ and pre-hemadsorber pressure [mmHg] = 0.6342 × MAP [mmHg] + 8.413 (R-square 0.9978) for the Oxiris^®^ hemadsorber, respectively (Figure 3).

Post-CytoSorb^®^ hemadsorber pressures also showed significant differences (*p* < 0.05) between the experimental steps, comparing an MAP of 45 mmHg to MAPs of 75 or 85 mmHg, respectively. Post-Oxiris^®^ hemadsorber pressures significantly differed between MAPs of 45 mmHg or 55 mmHg to an MAP of 85 mmHg. Nevertheless, post-hemadsorber pressures did not exceed maximum values of 22 ± 2 mmHg for the CytoSorb^®^ or 18.8 ± 4.8 mmHg for the Oxiris^®^ device.

## 4. Discussion

Hemoperfusion involves the passage of blood through a hemofilter in which mediators are adsorbed to a membrane surface or through a sorbent-containing cartridge. Adsorption is mostly utilized in combination with hemodialysis (HD) or extracorporeal membrane oxygenation (ECMO). Our data indicate that both the CytoSorb^®^ and the Oxiris^®^ systems can be used as independent therapeutic entities via a femorofemoral arteriovenous shunt. Shunt circulation resulted in sufficient blood flows through both devices over a wide range of mean arterial blood pressures from 45 to 85 mmHg. Lowest blood flow at an MAP of 45 mmHg was 194 ± 34 mL/min for the CytoSorb^®^ system and 203 ± 17 mL/min for the Oxiris^®^ system, well above the minimum flows recommended by the manufacturers of >100–150 mL/min [12,13]. We found a linear regression between blood flow through both devices and MAP. Pre-absorber pressures increased in linear regression with increasing MAP. The experiments further suggest that pEHAT is safe as none of the animals suffered from major or minor complications.

The predominant indication for hemadsorption in critical care is the removal of proinflammatory cytokines during septic shock and hyperinflammation. A case series showed that the combination of continuous renal replacement therapy (CRRT) and HA was associated with rapid hemodynamic stabilization and decrease in serum lactate [6]. In a study of twenty patients with refractory septic shock, early hemadsorption within twelve hours after the start of shock therapy significantly reduced noradrenalin, improved lactate clearance, and led to shock reversal in 65% of the patients [14]. A retrospective analysis of 116 patients found a significantly decreased observed versus expected 28-day all-cause mortality [6]. Initiation of therapy within 24 h of septic shock might be the key, as hemadsorption did not reduce mortality or lower plasma IL-6 levels in the presence of multiple organ failure [4]. This was further emphasized in a case series showing that hemodynamic stabilization and a reduction in blood lactate levels was more pronounced in patients in whom therapy started within 24 h after sepsis diagnosis. A poor response was observed in patients with delayed therapy [5]. These findings may be related to the fact that antigenic stimulation of naïve CD8+ T cells commits them to an instructive developmental program that does not require further antigenic stimulation and does not cease until memory CD8+ T cell formation [15].

The decisive asset of hemadsorption via an arteriovenous shunt is that it offers the flexibility to achieve early initiation of hemadsorption without the need for additional devices. In addition, it might be also used for the removal of different toxins, e.g., bilirubin, bile acids, free hemoglobin, myoglobin, drugs, etc. [16].

We observed adequate blood flows through both HA devices over a wide range of MAPs, which we believe are representative for clinical practice. Even at a very low MAP of 45 mmHg, the measured blood flows were above the minimum recommended flow rates and higher than what is usually achieved during CRRT. Moreover, the utilized setup is easily clinically applicable. Placement of an arteriovenous shunt was utilized for many years in extracorporeal lung assist devices (avECLA) in patients with severe acute respiratory distress [17,18,19]. A similar arteriovenous setup using smaller cannulas was chosen and could be safely and rapidly established by intensivists.

Nevertheless, we only utilized healthy animals, and future investigations are necessary in order to address the effectivity of cytokine elimination in man as well as clinical applicability of extracorporeal HA.

Furthermore, blood coagulation factors should be closely monitored during anticoagulation, as well as blood flow to the extremities, e.g., with additional pulse oximetry, especially if the experiment is conducted in sepsis and transferred to humans.

## 5. Conclusions

Pumpless extracorporeal hemadsorption technique is feasible and sufficient blood flow through the HA could be maintained even with low blood pressures mimicking septic shock. Future studies should evaluate if an early hemadsorption of proinflammatory cytokines with pEHAT may prevent a further organ dysfunction during septic shock with a hyperinflammatory response syndrome.

## Figures and Tables

**Table 1:** Systemic hemodynamic parameters.

	Baseline	MAP45	MAP55	MAP65	MAP75	MAP85	*p*-Value
**HR (L/min)**							
CytoSorb^®^	92 ± 6.2	82.7 ± 7.4	82.3 ± 4.4	84.3 ± 4.1	91.5 ± 7.5	103 ± 6.4	* 0.0011
Oxiris^®^	95.8 ± 20.9	91.8 ± 19.9	92.9 ± 13.7	90.9 ± 9.3	86.1 ± 8.3	89.9 ± 18.6	0.73
**CI (L/min/m^2^)**							
CytoSorb^®^	4.3 ± 0.2	3.7 ± 0.8	3,7 ± 0.8	3.9 ± 0.8	4.1 ± 0.5	4.4 ± 0.5	* 0.04
Oxiris^®^	4.4 ± 1	4.6 ± 0.7	4.2 ± 0.6	4.1 ± 1.5	3.8 ± 0.4	4.8 ± 0.9	0.41
**PAP (mmHg)**							
CytoSorb^®^	24 ± 2.3	19.9 ± 5.3	24.8 ± 1.6	23.8 ± 1.5	25.3 ± 1.5	26 ± 2.2	0.03
Oxiris^®^	24.6 ± 1.3	17.5 ± 3.2	20.8 ± 2.0	21.7 ± 1.1	23.3 ± 2.8	26.7 ± 1.1	* <0.0001
**CVP (mmHg)**							
CytoSorb^®^	13.1± 1.2	13.3 ± 2.9	15.5 ± 2.6	14.8 ± 2.6	13.4 ± 1.2	13.4 ± 0.5	0.45
Oxiris^®^	11.1 ± 2.2	7.3 ±1.7	9. 7 ± 2.1	10.9 ± 4.1	10.7 ± 2.1	12.5 ± 1.7	* 0.03

HR = heart rate; MAP = mean arterial pressure; CI = cardiac index; PAP = pulmonary artery pressure; CVP = central venous pressure. The *p*-value represents the overall probability of a type I error in the repeated measures ANOVA; * *p*-values < 0.05 were considered significantly different. In Bonferroni multiple comparison post hoc testing, no significant differences were found between the different MAP steps regarding CI or PAP for the CytoSorb^®^ device, respectively.

**Table 2:** Hemadsorber pressure and blood flow.

	Baseline	MAP45	MAP55	MAP65	MAP75	MAP85	*p*-Value
**Blood Flow (mL/min)**							
CytoSorb^®^	0	194.2 ± 33.5	222.9 ± 32.6	265.7 ± 22.2	312.5 ± 31.6	360.7 ± 39.9	<0.0001
Oxiris^®^	0	202.8 ± 16.8	237.4 ± 23.7	266.5 ± 24.5	316.4 ± 9.5	326.7 ± 14.1	<0.0001
**Pre-Hemadsorber Pressure (mmHg)**							
CytoSorb^®^	0	37.4 ± 1.7	44.3 ± 2.6	50 ± 2.4	58.1 ± 3.9	65.3 ± 5	<0.0001
Oxiris^®^	0	36.7 ± 3.4	43.4 ± 2.6	49.7 ± 3.5	56.7 ± 3.8	61.8 ± 4.0	<0.0001
**Post-Hemadsorber Pressure (mmHg)**							
CytoSorb^®^	0	14.5 ± 3.7	18.9 ± 1.8	18.9 ± 0.6	20 ± 1.4	22 ± 2	<0.0001
Oxiris^®^	0	11.8 ± 1.7	12.8 ± 2.4	15.8 ± 3.2	15.3 ± 3.8	18.8 ± 4.8	<0.0001

The *p*-value represents the overall probability of a type I error in the repeated measures ANOVA. There were significant differences in the repeated measures ANOVA (*p* < 0.0001) between the different MAP steps in all three parameters for both devices, respectively.

## Data Availability

The datasets used and/or analyzed during the current study are available from the corresponding author on reasonable request.

## Abbreviations

CO	Cardiac output
CRRT	Continuous renal replacement therapy
CVVH	Continuous veno-venous hemofiltration
CVP	Central venous pressure
ECMO	Extracorporeal membrane oxygenation
HA	Extracorporeal hemadsorption
HD	Hemodialysis
HP	Hemoperfusion
MAP	Mean atrial pressure
PAP	Pulmonary arterial pressure
PEI	Polyethyleneimine

## References

[1] Kovacs J. (2020). Hemoadsorption in Critical Care—It is a Useful or a Harmful Technique?. J. Crit. Care Med..

[2] Ankawi G., Xie Y., Yang B., Xie Y., Xie P., Ronco C. (2019). What Have We Learned about the Use of Cytosorb Adsorption Columns?. Blood Purif..

[3] Chen G., Zhou Y., Ma J., Xia P., Qin Y., Li X. (2020). Is there a role for blood purification therapies targeting cytokine storm syndrome in critically severe COVID-19 patients?. Ren. Fail..

[4] Schadler D., Pausch C., Heise D., Meier-Hellmann A., Brederlau J., Weiler N., Marx G., Putensen C., Spies C., Jörres A. (2017). The effect of a novel extracorporeal cytokine hemoadsorption device on IL-6 elimination in septic patients: A randomized controlled trial. PLoS ONE.

[5] Kogelmann K., Jarczak D., Scheller M., Druner M. (2017). Hemoadsorption by CytoSorb in septic patients: A case series. Crit. Care.

[6] Brouwer W.P., Duran S., Kuijper M., Ince C. (2019). Hemoadsorption with CytoSorb shows a decreased observed versus expected 28-day all-cause mortality in ICU patients with septic shock: A propensity-score-weighted retrospective study. Crit. Care.

[7] Rieder M., Wengenmayer T., Staudacher D., Duerschmied D., Supady A. (2020). Cytokine adsorption in patients with severe COVID-19 pneumonia requiring extracorporeal membrane oxygenation. Crit. Care.

[8] Rieder M., Duerschmied D., Zahn T., Lang C., Benk C., Lother A., Paul B., Christoph B., Tobias W., Dawid S. (2021). Cytokine Adsorption in Severe Acute Respiratory Failure Requiring Veno-Venous Extracorporeal Membrane Oxygenation. Asaio J..

[9] Fiedler M.O., Reyher C., Kalenka A., Rolfes C., Lotz C., Muellenbach R.M. (2021). Pumpless Extracorporeal Hemadsorption Technique: A New Method for Early Cytokine Elimination?. Blood Purif..

[10] Napp L.C., Ziegeler S., Kindgen-Milles D. (2019). Rationale of Hemoadsorption during Extracorporeal Membrane Oxygenation Support. Blood Purif..

[11] Monard C., Rimmelé T., Ronco C. (2019). Extracorporeal Blood Purification Therapies for Sepsis. Blood Purif..

[12] https://www.baxter.com/oxiris-critical-care.

[13] https://cytosorb-therapy.com/en/the-adsorber/.

[14] Friesecke S., Stecher S.S., Gross S., Felix S.B., Nierhaus A. (2017). Extracorporeal cytokine elimination as rescue therapy in refractory septic shock: A prospective single-center study. J. Artif. Organs.

[15] Kaech S.M., Ahmed R. (2001). Memory CD8+ T cell differentiation: Initial antigen encounter triggers a developmental program in naïve cells. Nat. Immunol..

[16] Ankawi G., Neri M., Zhang J., Breglia A., Ricci Z., Ronco C. (2018). Extracorporeal techniques for the treatment of critically ill patients with sepsis beyond conventional blood purification therapy: The promises and the pitfalls. Crit. Care.

[17] Muellenbach R.M., Kredel M., Kuestermann J., Klingelhoefer M., Schuster F., Wunder C., Kranke P., Roewer N., Brederlau J. (2009). Combining “open-lung” ventilation and arteriovenous extracorporeal lung assist: Influence of different tidal volumes on gas exchange in experimental lung failure. Med. Sci. Monit..

[18] Muellenbach R.M., Kredel M., Wilhelm J., Küstermann J., Fink L., Siebenliest G., Klosterhalfen B., Foerster C.Y., Kranke P., Wunder C. (2010). High-frequency oscillation combined with arteriovenous extracorporeal lung assist reduces lung injury. Exp. Lung Res..

[19] Muellenbach R.M., Wunder C., Nuechter D.C., Smul T., Trautner H., Kredel M., Roewer N., Brederlau J. (2007). Early treatment with arteriovenous extracorporeal lung assist and high-frequency oscillatory ventilation in a case of severe acute respiratory distress syndrome. Acta Anaesthesiol. Scand..

